# Supplementary material for: Influence of Genetic Variations in miRNA and Genes Encoding Proteins in the miRNA Synthesis Complex on Toxicity of the Treatment of Pediatric B-Cell ALL in the Brazilian Amazon
Source: Int J Mol Sci. 2023 Feb 23;24(5):4431. doi: 10.3390/ijms24054431 (PMC10003057; doi:10.3390/ijms24054431)
Supplement: Supplementary file 1 [file ijms-24-04431-s001.zip › ijms-2057633-supplementary.pdf]

Supplementary Table S1. Single nucleotide variants (SNVs) of the 25 marker custom panel in miRNA genes and in genes encoding proteins essential for the synthesis of miRNAs.

GENE	ID SNV	Allelic Variation	MAF [#]	Chromosomal location (GH38) *
DROSHA	rs639174	C/T	T: 0.35	Chr.5: 31433540
C5orf22	rs10035440	C/T	C: 0.18	Chr.5: 31539356
MIR323B	rs56103835	C/T	C: 0.18	Chr.14: 101056219
MIR2053	rs10505168	C/T	C: 0.35	Chr.8: 112643523
DROSHA	rs3805500	G/A	G: 0.40	Chr.5: 31462870
MIR300	rs12894467	C/T	C: 0.49	Chr.14: 101041390
MIR146A	rs2910164	C/G	C: 0.29	Chr.5: 160485411
MIR196A2	rs11614913	C/T	T: 0.38	Chr.12: 53991815
MIR149	rs2292832	T/C	T: 0.41	Chr.2: 240456086
AGO1	rs636832	A/G	A: 0.12	Chr.1: 35897874
MIR200A	rs9660710	C/A	A: 0.07	Chr.1: 1163962
KRT86	rs3660	C/G	G: 0.41	Chr.12: 52286153
MIR219A1	rs213210	A/G	A: 0.23	Chr.6: 33208047
MIR499A	rs3746444	A/G	G: 0.18	Chr.20: 34990448
MIR608	rs4919510	C/G	G: 0.35	Chr.10: 100975021
MIR605	rs2043556	C/T	T: 0.27	Chr.10: 51299646
EFNA1	rs12904	A/G	A: 0.41	Chr.1: 155134221
MIR938	rs2505901	C/T	C: 0.49	Chr.10: 29602392
CSK	rs2168518	A/G	A: 0.24	Chr.15: 74788737
MIRLET7A1	rs10739971	A/G	A: 0.20	Chr.9: 94175398
MIR219A1	rs107822	C/T	G: 0.47	Chr.6: 33207798
NSRP1	rs6505162	A/C	C: 0.41	Chr.17: 30117165
MIR604	rs2368392	A/G	A: 0.29	Chr.10: 29545074
CTDSPL	rs7372209	C/T	T: 0.25	Chr.3: 37969217
MIR100HG	rs1834306	A/G	A: 0.38	Chr.11: 122152479

*** References to chromosomal locations were obtained from the GH38 version of the human genome obtained from the Human Genome Project; [#] Minor allele frequency.**

Picture Supplementary S1. Hardy-Weinberg Equilibrium analysis of the investigated variants.

**Variants**	**Hardy-Weinberg equilibrium (*p*-value)**
rs12904	0.4378
rs636832	0.8625
rs12894467	0.2769
rs3746444	**0.0274***
rs213210	0.4209
rs107822	0.8371
rs4919510	0.8526
rs3805500	0.0880
rs10035440	0.2212
rs1834306	0.5554
rs2292832	0.2221
rs6505162	**0.0005***
rs2043556	**0.0269***
rs3660	**<0.0001***
rs2168518	0.6287
rs2910164	**0.0238***
rs2368392	**0.0033***
rs2505901	0.9222
rs639174	0.6357
rs7372209	0.1576
rs10505168	0.1567
rs9660710	0.5329
rs10739971	**0.0002***
rs11614913	0.2298
rs56103835	**0.0430***

* Genetic Variants in Hardy Weinberg Disequilibrium
